# Cytotoxic Indole Alkaloid 3*α*-Acetonyltabersonine Induces Glioblastoma Apoptosis via Inhibition of DNA Damage Repair

**DOI:** 10.3390/toxins9050150

**Published:** 2017-04-28

**Authors:** Yuan Li, Yunli Zhao, Xia Zhou, Wei Ni, Zhi Dai, Dong Yang, Junjun Hao, Lin Luo, Yaping Liu, Xiaodong Luo, Xudong Zhao

**Affiliations:** 1 Key Laboratory of Bioactive Peptides of Yunnan Province/Key Laboratory of Animal Models and Human Disease Mechanisms of Chinese Academy of Sciences, Kunming Institute of Zoology, Chinese Academy of Sciences, 21 Qingsong Road, Kunming 650203, Yunnan, China; liyuan@mail.kiz.ac.cn (Y.L.); zhouxia@mail.kiz.ac.cn (X.Z.); daizhi@mail.kiz.ac.cn (Z.D.); yangdong@mail.kiz.ac.cn (D.Y.); 2 State Key Laboratory of Phytochemistry and Plant Resources in West China, Kunming Institute of Botany, Chinese Academy of Sciences, 132 Lanhei Road, Kunming 650201, China; zhaoyunli@mail.kib.ac.cn (Y.Z.); liuyaping@mail.kib.ac.cn (Y.L.); 3 Kunming College of Life Science, University of Chinese Academy of Sciences, 19 Qingsong Road, Kunming 650203, China; 4 Graduate School, University of Chinese Academy of Sciences, 19 Yuquan Road, Beijing 100049, China; 5 Division of Science and Technology, Kunming University of Science and Technology, 727 South Jingming Road, Kunming 650500, China; 6 Department of Neurosurgery, Tumor Hospital of Yunnan Province, 519 Kunzhou Road, Kunming 650000, China; niweikm@163.com (W.N.); luolvip@163.com (L.L.); 7 State Key Lab of Genetic Resources and Evolution, Kunming Institute of Zoology, Chinese Academy of Sciences, 21 Qingsong Road, Kunming 650203, China; haojunjun@mail.kiz.ac.cn; 8 Kunming Primate Research Center, Chinese Academy of Sciences, 32 East Jiaochang Road, Kunming 650223, China

**Keywords:** *melodinus suaveolens*, indole alkaloid, 3*α*-acetonyltabersonine, glioblastoma, cell apoptosis, DNA damage repair

## Abstract

Cytotoxic indole alkaloids from *Melodinus suaveolens*, which belongs to the toxic plant family Apocynaceae, demonstrated impressive antitumor activities in many tumor types, but less application in glioblastoma, which is the lethal brain tumor. In the present study, we reported the anti-glioblastoma activity of an indole alkaloid, 3*α*-acetonyltabersonine, which was isolated from *Melodinus suaveolens*. 3*α*-acetonyltabersonine was cytotoxic to glioblastoma cell lines (U87 and T98G) and stem cells at low concentrations. We verified 3*α*-acetonyltabersonine could suppress tumor cell proliferation and cause apoptosis in glioblastoma stem cells (GSCs). Moreover, detailed investigation of transcriptome study and Western blotting analysis indicated the mitogen activated protein kinase (MAPK) pathway was activated by phosphorylation upon 3*α*-acetonyltabersonine treatment. Additionally, we found 3*α*-acetonyltabersonine inhibited DNA damage repair procedures, the accumulated DNA damage stimulated activation of MAPK pathway and, finally, induced apoptosis. Further evidence was consistently obtained from vivo experiments on glioblastoma mouse model: treatment of 3*α*-acetonyltabersonine could exert pro-apoptotic function and prolong the life span of tumor-bearing mice. These results in vitro and in vivo suggested that 3*α*-acetonyltabersonine could be a potential candidate antitumor agent.

## 1. Introduction

Glioblastoma is the most common malignant brain tumor with average only 14 months survival time and constitutes about 50% of all brain parenchymal tumors. Even though maximal therapy have been done in clinic, effects remains to be improved. Seeking new and effective compounds applied in chemotherapy is an essential therapy strategy and is urgently needed. 

Natural products are valuable sources for drug discovery. They are used as lead compounds to obtain analogs with different biological activities, in particular antitumor agents [1,2,3,4]. Monoterpenoid indole alkaloids, which are made by condensing tryptophan with secologanin, are attractive resources for drug development because of their diverse structures and promising pharmacological activities [5]. Vinca alkaloids, which structurally belong to the bisindole group, are well known for their antitumor bioactivities. Natural products from this group, such as vinblastine [6], vincristine [7], vindesine [8] and their structurally modified compounds, are currently commercial drugs and are used in the clinic to treat cancers in many countries [9]. Searching for optimized or simplified indole alkaloids has been the spotlight of antitumor drug discovery for several years. In our continual search for natural antitumor products from medicinal plants, indole alkaloids were found to be the most potent candidates in a cytotoxicity bioassay against five human cancer cell lines [10,11,12,13,14,15,16,17,18,19,20,21,22,23,24,25,26,27,28,29]. Among these cytotoxic indoles, 3*α*-acetonyltabersonine, an aspidosperma-type monoterpenoid indole alkaloid, structurally similar to vindoline, showed the best bioactivity, with IC_50_ values of 0.2–0.6 μM against human myeloid leukemia HL-60 cells, hepatocellular carcinoma SMMC-7721 cells, lung cancer A549 cells, breast cancer MCF-7 cells, and colon cancer SW480 cells. Preliminary structure-activity relationship (SAR) investigation indicated that the acetonyl moiety at C-3 of tabersonine increased cytotoxic bioactivity, while the hydroxyl substituent decreased it remarkably [11]. 

In this report, we investigated more detail on the bioactivity of 3*α*-acetonyltabersonine. Besides the cytotoxicity against the above-mentioned five tumors, 3*α*-acetonyltabersonine showed significant cytotoxicity against glioblastoma, more meaningfully, 3*α*-acetonyltabersonine exerted better cytotoxicity against GSCs than glioblastoma cell lines. GSCs were one of the first verified stem cells isolated from solid tumor in 2003 [30]. Cancer stem cells are a small population cells in cancers but important for cancer initiation, maintenance and metastasis [31,32]. Unfortunately, cancer stem cells are more resistant than other cancer cells to current cancer therapies, which often results in tumor relapse after initial tumor regression. Therefore, the drugs targeting cancer stem cells may have better therapeutic effect on cancers. Due to the significant antitumor effect of 3*α*-acetonyltabersonine, the cytotoxic mechanism of the compounds was investigated, and found that 3*α*-acetonyltabersonine induced cell apoptosis by triggering MAPK phosphorylation through inhibition of the DNA damage repair process. Furthermore, 3*α*-acetonyltabersonine could also induce cell apoptosis in vivo and prolong the survival span of glioblastoma mouse model. 

## 2. Results

### 2.1. 3α-Acetonyltabersonine Showed Antitumor Cytotoxicity in Glioblastoma

**Figure 1:**
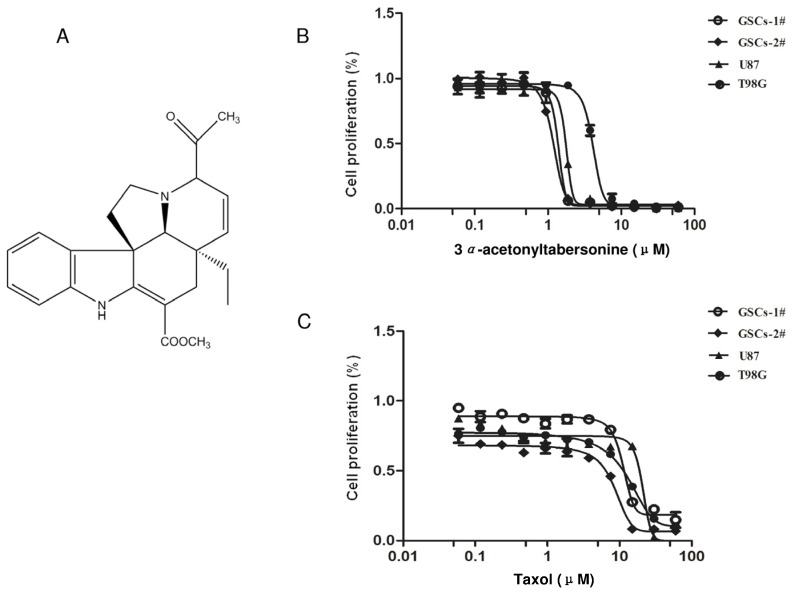
Identification of 3*α*-acetonyltabersonine as a potent antitumor compound in glioblastoma cell lines and GSCs: (**A**) chemical structure of 3*α*-acetonyltabersonine; and (**B**,**C**) MTS assay of 3*α*-acetonyltabersonine and taxol (positive control) on GSCs (GSCs-1# and GSCs-2#) and glioblastoma cell lines (U87 and T98G).

To determine whether 3*α*-acetonyltabersonine (Figure 1A) could be cytotoxic to glioblastoma, two GSCs isolated from clinic samples and two widely used cell lines, U87 and T98G, were used to conduct [3-(4,5-dimethylthiazol-2-yl)-5-(3-carboxymethoxyphenyl)-2-(4-sulfophenyl)-2H-tetrazolium] (MTS) assay. The IC_50_ values of GSCs-1# and GSCs-2# were 1.30 μM and 1.13 μM, and the IC_50_ values of glioblastoma cell lines U87 and T98G were 1.7 μM and 4.3 μM, respectively (Figure 1B). Here, we employed taxol as a positive control. The IC_50_ values of taxol on the four cells, especially on GSCs, were not notable (Figure 1C). Such results demonstrated that 3*α*-acetonyltabersonine exerted considerable cytotoxicity against glioblastoma. 

### 2.2. 3α-Acetonyltabersonine Suppressed Proliferation and Induced Apoptosis in GSCs

**Figure 2:**
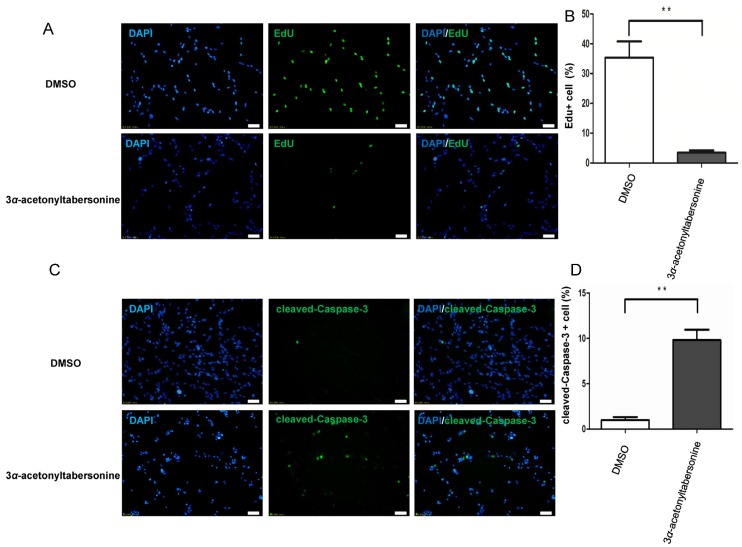
3*α*-acetonyltabersonine suppressed cell proliferation and induced apoptosis of GSCs: (**A**) The number of proliferative cells labeled by EdU was significantly different between the 3*α*-acetonyltabersonine treatment group and the group lacking 3*α*-acetonyltabersonine (0.25 μM, 24 h) (bar = 50 μm). Quantitative analysis on the data of EdU+ cell percent presented in panel (**B**). (**C**) Immunofluorescence images labeled by cleaved-Caspase-3 staining. The cells were incubated without or with 3*α*-acetonyltabersonine (2 μM, 48 h). Quantitative analysis on the data of cleaved-Caspase-3 + cell percent presented in panel (**D**). Blue and green fluorescence represented DAPI and cleaved-Caspase-3, respectively (bar = 50 μm). ** *p* < 0.01 versus control group.

**Figure 3:**
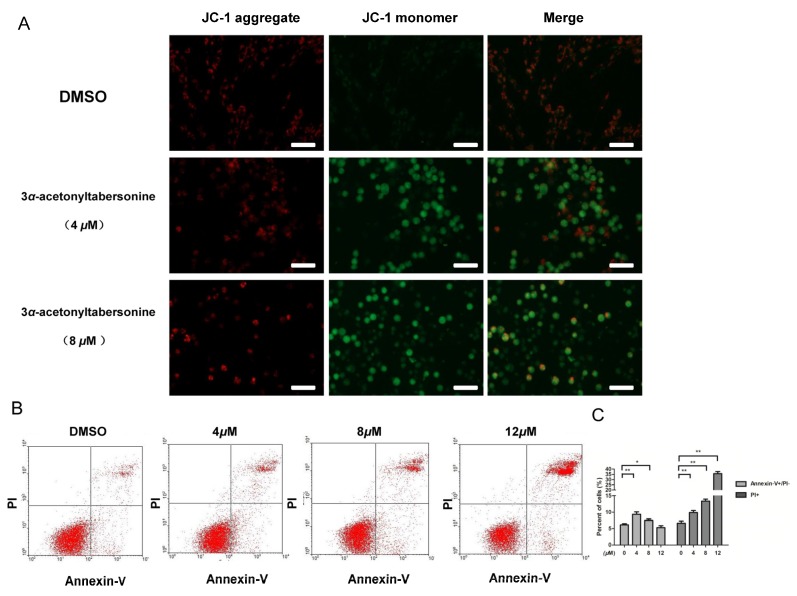
Cellular apoptosis induced by 3*α*-acetonyltabersonine in GSCs. (**A**) Morphological photographs of JC-1 staining in cells that were treated with different concentrations of 3*α*-acetonyltabersonine or vehiclefor 12 h. The increasing ratio of green fluorescence/red fluorescence indicates the decrease in MMP, which is symbolic of cellular apoptosis (bar = 50 μm). (**B**) Apoptosis analysis by FCM verified again that3*α*-acetonyltabersonine induced cellular apoptosis. The cells were incubated with DMSO or three concentration gradients of 3*α*-acetonyltabersonine for 16 h. Quantitative analysis on the data presented in panel (**C**). * *p* < 0.05 and ** *p* < 0.01 versus control group.

The considerable cytotoxicity of 3*α*-acetonyltabersonine led us to conduct further investigation. Firstly, we tested whether 3*α*-acetonyltabersonine suppressed cell proliferation and/or induced apoptosis in vitro. 5-Ethynyl-2′-deoxyuridine (EdU) assay was utilized to examine DNA replication after 3*α*-acetonyltabersonine treatment, and the results are shown in Figure 2A. The amount of EdU+ cells in 3*α*-acetonyltabersonine-treated group was approximately 3.45%, but there were about 35.3% of EdU+ cell in the dimethyl sulfoxide (DMSO) group. EdU could instead of thymine in the DNA replication period incorporate into the newly replicated DNA, so EdU assay reflect the activity of DNA synthesis and cell proliferation. The percentages of EdU+ decreased significantly after 3*α*-acetonyltabersonine treatment, suggesting 3*α*-acetonyltabersonine inhibited cell proliferation. Meanwhile, 3*α*-acetonyltabersonine caused cell apoptosis dramatically, as shown by increased cleaved-Caspase-3 immunofluorescence (Figure 2C), JC-1 staining (Figure 3A) and Annexin-V/PI FCM (Figure 3B). JC-1 staining is indicative of mitochondrial membrane potential (MMP), an important indicator of cell viability. The bright red fluorescence of JC-1 aggregates could be observed in healthy cells, whereas apoptotic cells, which are in a state of mitochondrial dysfunction as indicated by MMP loss, display green fluorescence. In Figure 3A, the MMP of the control cells produced conspicuous red staining with minimal green fluorescence. However, in the 3*α*-acetonyltabersonine-treated group, a marked reduction in red fluorescence and an increase in green fluorescence were observed in the cells, indicating the loss of MMP and the induction of apoptosis. Caspase-3 is one of the most extensively studied apoptotic proteins and is regarded as a key effector in the apoptotic pathway. The expression of its active form, cleaved-Caspase-3, indicates that cells have entered into the late apoptotic process. As we present in Figure 2C, the ratio of apoptotic cells stained for cleaved-Caspase-3 in 3*α*-acetonyltabersonine treated cells was much greater than that of the DMSO-treated group, and the difference was significant. Annexin-V and PI label early apoptotic cells and late apoptotic cells, respectively. In the FCM analysis, the distribution of cells in the lower right and upper right quadrants indicated Annexin-V/PI+ cells, representing the cell populations in the early and late phases of the apoptotic process, respectively. Briefly, as shown in Figure 3B, 3*α*-acetonyltabersonine dramatically increased the proportion of apoptotic cells in a dose-dependent manner. 

### 2.3. Phosphorylation of the MAPK Signaling Pathway Was Modulated by the Activity of 3α-Acetonyltabersonine

**Figure 4:**
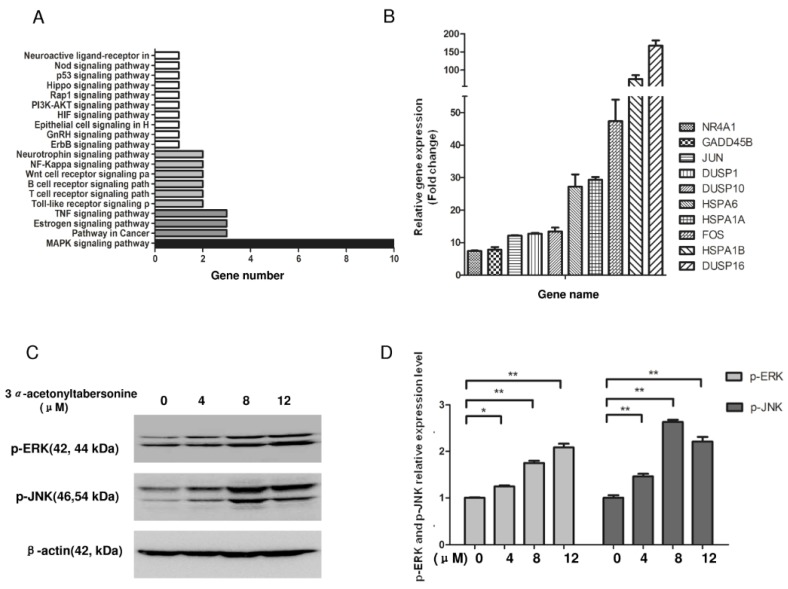
The MAPK pathway was activated via phosphorylation, and the genes of the pathway were upregulated after 3*α*-acetonyltabersonine treatment in GSCs. (**A**) The distribution of genes that significantly changed after exposure to 3*α*-acetonyltabersonine clustered mostly in the MAPK signaling pathway. All the signaling pathway statistics were based on the Kyoto Encyclopedia of Genes and Genomes (KEGG) database catalogue (http://www.kegg.jp/kegg/pathway.html). (**B**) The genes with significant changes within the MAPK pathway were verified by quantitative real-time PCR. (**C**) The activation levels of the ERK and JNK pathways were reflected directly by the expression levels of p-ERK and p-JNK. β-actin was used as the loading control. (**D**) The change of p-ERK and p-JNK were significant. Quantitative analysis of the data presented in panel (**D**).* p < 0.05 and ** p < 0.01 versus control group.

RNA-seq was performed to explore the pharmacological mechanism of this compound. Target sample and control sample were sequenced using an Illumina HiSeq 2000 platform in paired-end form over 101 bp. Then, a total of approximately 6 GB of raw sequence data were attained for each sample, with Q20 > 95% and Q30 > 89%, indicating high quality. The raw sequence reads were deposited at NCBI (Accession No. GSE95392). Cuffmerge was used to merge transcripts of the 3*α*-acetonyltabersonine-treated group and the control group. In total, 66 significant different genes (*q* value < 0.05) were identified by comparing the 3*α*-acetonyltabersonine-treated group with the control using Cuffdiff. As the graph indicates, these genes were mapped to the signaling pathway and were enriched in several signaling pathways, most notably the MAPK signaling pathway (Figure 4A). There were 10 genes, *Dusp16*, *Hspa1b*, *Fos*, *Hspa1a*, *Hspa6*, *Dusp10*, *Dusp1*, *Jun*, *Gadd45b*, and *Nr4a1*, which were upregulated in a cluster in the MAPK pathway. Then, the RNA-seq data were regularly validated by quantitative real-time PCR. Consistently, the mRNA expression level of the all genes were upregulated in the drug-treatment group (Figure 4B). The majority of the genes were mapped to the ERK and JNK pathways, the two main branches of the MAPK pathway, therefore, we also detected the expression levels of p-ERK and p-JNK , the active form of ERK and JNK, by Western blotting. As shown in Figure 4C,D, even at a low dose (4 μM), the level of the activation of p-JNK and p-ERK, especially that of p-JNK, was considerable.

### 2.4. 3α-Acetonyltabersonine Inhibited DNA Damage Repair

**Figure 5:**
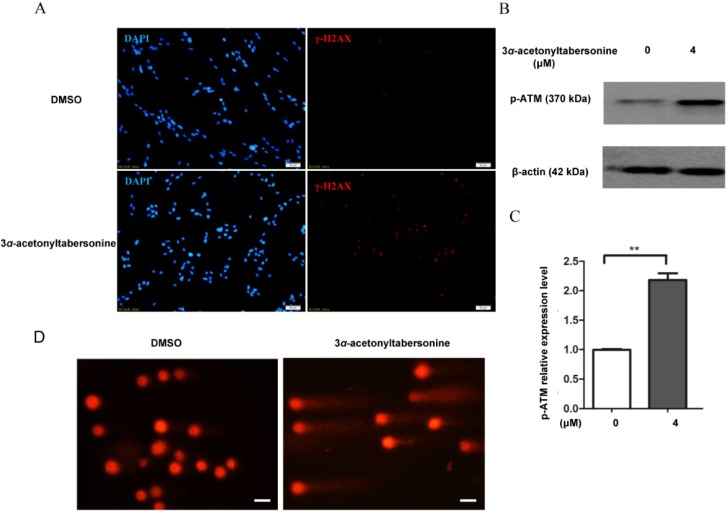
GSCs apoptosis induced by 3*α*-acetonyltabersonine may relate to DNA damage. (**A**) The expression of γ-H2AX was higher in 3*α*-acetonyltabersonine-treated cells (bar = 50 μm). (**B**) Cells treated with 3*α*-acetonyltabersonine showed upregulation of p-ATM, indicating that DNA damage occurred. (**C**) The quantitative analysis of the expression level of the p-ATM protein before and after 3*α*-acetonyltabersonine treatment showed the level of p-ATM significantly increased under the drug added condition. (**D**) A comet assay was used as a measurement for DNA lesions. Longer tails, a typical feature of DNA fragments, appeared more frequently and obviously after drug treatment than the control (bar = 50 μm). Quantitative analysis of the data presented in panel (**C**). ** p < 0.01 versus control group.

Many studies have reported that the MAPK pathway might respond to DNA-damaging agents [33,34], then, γ-H2AX and ATM, the markers of DNA damage, were employed to determine whether DNA damage occurred during 3*α*-acetonyltabersonine-induced apoptosis. As shown in Figure 5A, γ-H2AX expression in the 3*α*-acetonyltabersonine-treated group was stronger than that in the vehicle control, indicating increased DNA damage caused by 3*α*-acetonyltabersonine. Likewise, similar results were observed in the expression level of p-ATM protein, the activated form of ATM, as probed by Western blotting (Figure 5B,C). In addition, a comet assay, which is widely accepted as the criterion to assess DNA lesion status, was performed. The 3*α*-acetonyltabersonine-treated cells showed a long-tailed staining of fragmented DNA, while the staining in the control cells maintained an intact point (Figure 5D). The result suggested that cells exposed to 3*α*-acetonyltabersonine produced many nucleus fragments, which is indicative of the appearance of DNA lesions. 

**Figure 6:**
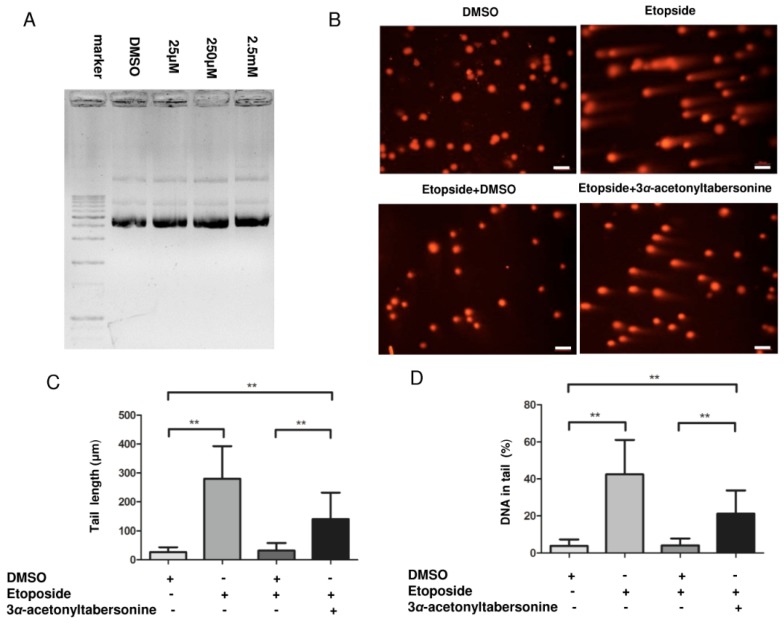
The inhibition of DNA damage repair was the actual target of 3*α*-acetonyltabersonine in GSCs. (**A**) Analysis of DNA damage in vitro via a plasmid cleavage assay converted to a visual electrophoretogram. From top to bottom, the bands were nicked DNA, linearized DNA and supercoiled DNA, and the three bands of all groups were not obviously different. (**B**) Four groups of parallel experiments demonstrated the ability of 3*α*-acetonyltabersonine to inhibit GSCs recovery from DNA damage. Effects of drug were evaluated by a comet assay. The Etoposide+DMSO group demonstrated the recovery effect. After 6 h of recovery time, the nucleus returned to its regular round shape, which meant that reduction of etoposide cytoxocity. If etoposide was replaced by 3*α*-acetonyltabersonine, the recovery did not occur. The DMSO group and the etoposide group were used as negative and positive controls, respectively (bar = 100 μm). (**C**,**D**) The degrees of DNA damage of the four groups were estimated via tail length and the percent DNA in the tail index. Statistical results provided quantitative proof for the DNA damage and recovery process. Quantitative analysis of the data presented in panels (**C**) and (**D**). ** *p* < 0.01 versus control group.

Plasmid cleavage assay that could recapitulate the DNA cleavage status was performed to better understand the details in DNA breaks by 3*α*-acetonyltabersonine [35]. Then the plasmid was incubated with different concentrations of 3*α*-acetonyltabersonine ranging from 25 μM to 2.5 mM. The electrophoresis technique was used to check the plasmid integrity, three types of bands: nicked DNA, linearized DNA and supercoiled DNA, which represent DNA single-strand breaks (ssbs), double-strand breaks (dsbs) and no breaks, respectively, were detected. As shown in Figure 6A, the ratio of the three bands did not appear to change, which indicated that the intact DNA did not obvious transform into single-strand or double-strand DNA. The bands of ssbs and dsbs were weak and there were no obvious differences among the four groups. These results indicated 3*α*-acetonyltabersonine treatment did not directly attack DNA. The DNA damage verified in this paper was not mainly the result of direct attack on DNA, so the possible reason was the DNA repair process had been inhibited. To test this hypothesis, we conducted a group of comet assay. The group treated by solvent retained their original appearance, while the group exposed to etoposide exhibited a large amount of fragmented DNA. Two other groups were first treated with etoposide, then after with DMSO or 3*α*-acetonyltabersonine. The cells in the group replaced by DMSO recovered well, as shown by the loss of tail staining, but, in cells in the group replaced by 3*α*-acetonyltabersonine, DNA fragments remained (Figure 6B–D). This phenomenon suggested that 3*α*-acetonyltabersonine suppressed the DNA damage recovery process and led to apoptosis.

### 2.5. 3α-Acetonyltabersonine Increased the Lifespan in Glioblastoma Mouse Model

**Figure 7:**
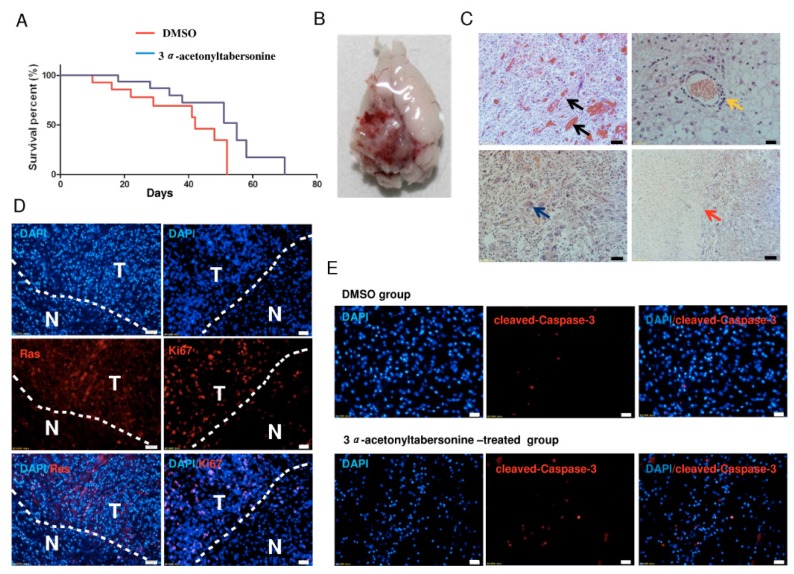
The toxicity effect of 3*α*-acetonyltabersonine tested in vivo. (**A**) The Kaplan–Meier survival curves of C57BL/6 mice injected with pTomo-Ras-sip53 lentivirus and the subsequent drug delivery effect (*n* = 9 for each group). (**B**) Surface image of a glioblastoma brain from the mouse model after infection with pTomo-Ras-sip53 lentivirus. (**C**) The microscopic appearance of pathologic glioblastoma features, including blood vessel hyperplasia and microvascular proliferation (black arrow), endothelial cell proliferation surrounding the vascellum (yellow arrow), giant cell formation (blue arrow), the border of normal tissue and a highly dense cellular region (red arrow) (bar = 50 μm). (**D**) Gene expression detected by immunohistochemistry of Flag-tagged H-Ras (bar = 50 μm) and Ki67 (bar = 20 μm) in the area between the tumor (T) and normal (N) tissues. (**E**) The examination of cleaved-Caspase-3 expression in the 3*α*-acetonyltabersonine-treated group and the control group (bar = 50 μm).

Mouse models recapitulating human features of disease are important for cancer research and therapy development. Previously reported mouse glioblastoma model created by constitutively expressing active H-RasV12 and silencing p53 [36] was used to evaluate antitumor effect of 3*α*-acetonyltabersonine in this study. The average lifespan of mice in drug-administration group was 44.8 days, prolonged about 38% compared with the average lifespan of 32.5 days in DMSO treatment group (Figure 7A). Anatomical observation of the tumor-bearing mice exhibited typical glioblastoma histopathological features (Figure 7B–D). Immunohistochemical staining of representative brain sections from the 3*α*-acetonyltabersonine-treated group expressed more of the apoptotic marker cleaved-Caspase-3 than the control group (Figure 7E). 

## 3. Discussion

Widely used antitumor indole alkaloids, such as vincristine, exert a wide-spectrum antitumor effect on leukemia, hepatocarcinoma, colorectal, prostate, lung, and breast cancers [37,38,39,40,41,42], but few reported in glioblastoma. Chemotherapies primarily exert their antitumor effects by triggering apoptosis in cancer cells [43]. Herein, we proposed the pro-apoptotic function of 3*α*-acetonyltabersonine in GSCs at different stages and perspective, because chemotherapeutics, if they can eliminate glioblastoma stem cell, would be an important target for antitumor therapy. Moreover, 3*α*-acetonyltabersonine could inhibit glioblastoma stem cell proliferation. Some compounds, such as disulfiram, which inhibited the proliferation of glioblastoma stem cell and improved prognosis in GBM patients [44], have verified the feasibility of GSCs-target strategy. Therefore, based on this information, the potency of 3*α*-acetonyltabersonine against GSCs and cell lines, compared to others, represented impressive progress of such compounds, warranting further research on this compound.

JNK and ERK converges apoptotic signals from various stimuli and can be activated through phosphorylation in response to these signals. They regulate a multitude of processes in extrinsic and intrinsic pathways, which lead to the upregulation of pro-apoptotic components and the downregulation of anti-apoptotic components; thus, JNK and ERK play a central role in apoptotic pathways [45]. The mitochondrion has been thought to play a critical role in the intrinsic apoptotic pathway, partly because of the maintenance of the MMP. In the present study, 3*α*-acetonyltabersonine treatment activated both the JNK and ERK pathways and induced apoptosis, accompanied by the dissipation of the MMP in a concentration-dependent manner, which suggested that 3*α*-acetonyltabersonine might induce apoptosis predominately by a mitochondria-dependent mechanism. 

*Fos* and *Jun* were upregulated after 3*α*-acetonyltabersonine treatment from the RNA-seq and quantitative real-time PCR results. Additionally, FOS and JUN make up the dimeric transcription factor AP-1, which can modulate various cellular events, including apoptosis. Extracellular signaling, a variety of environmental stresses and chemicals can stimulate MAPK activation; soon afterwards, the activated MAPK translocates into the nucleus and induces AP-1 activity [46]. Such result was noted in the cell death induced by vinblastine in KB-3 cell [34]. Our findings on activation of ERK and JNK and upregulation of *Fos* and *Jun* (AP-1) expression, suggested that transcription factors, such as AP-1, might participate in the apoptosis process modulated by MAPK signaling pathway, in agreement with the general view. 

The mechanisms of indole alkaloid derivatives have been studied thoroughly. Vinblastine and vincristine cause cytotoxicity by binding on microtubulin and cause difficulty in mitosis. Camptothecin binds on the topoisomerase I (TOP1)-DNA complex, blocks DNA replication and finally induces cell apoptosis [47]. Some other indole alkaloids were observed to cause DNA breaks in cancer cell lines, but with limited investigation in detail [48]. Drugs destroying genome stability of cancer cell are considered important cancer therapeutics [49], thus 3*α*-acetonyltabersonine, which inhibited DNA lesion repair and the accumulation of DNA damage threatened on genome stability, is of great importance and enriches the antitumor mechanism in indole alkaloids.

## 4. Conclusions

In summary, 3*α*-acetonyltabersonine induced apoptosis by inhibition of DNA repair through MAPK pathway phosphorylation modulation in vitro and its anti-glioblastoma effect was also verified in vivo. Therefore, it could be a promising candidate agent against glioblastoma.

## 5. Materials and Methods 

### 5.1. Chemicals and Reagents 

3*α*-acetonyltabersonine was isolated from *M. suaveolens* and kept in the refrigerator at −20 °C [11]. Dulbecco’s modified Eagle’s medium (DMEM), DMEM/F12 medium, epidermal growth factor (EGF), basic fibroblast growth factor (bFGF), B27, NeuroMix, fetal bovine serum (FBS), penicillin-streptomycin (P/S), laminin and trypsin-EDTA were purchased from Life (Grand Island, NY, USA). TrypLE Express was purchased from Life (Copenhagen, CR, Denmark). SYBR Select Master Mix was purchased from Life (Austin, TX, USA). MTS solution was purchased from Promega (San Luis Obispo, CA, USA). Goat serum was purchased from Biosynthesis (Beijing, Beijing, China). Annexin-V/PI apoptotic assay kit was purchased from Neobioscience (Shenzhen, Guangdong, China). GelRed was purchased from Biotium (Fremont, CA, USA). Bovine serum albumin (BSA) was purchased from MP Biomedicals (Auckland, AL, New Zealand). Aqua-poly was purchased from Polysciences (Warrington, PA, USA). Immobilon western chemiluminescent horseradish peroxidase (HRP) substrate kit was purchased from Millipore (Billerica, MA, USA). Revert-Aid cDNA synthesis kit was purchased from Thermo Scientific (Vilnius, CR, Lithuania). JC-1 was purchased from Cayman (Ann Arbor, MI, USA). Click-iT EdU imaging Kits was purchased from Invitrogen (Carlsbad, CA, USA). Stereotaxic frame instruments and intracranial drug delivery was purchased from RWD (Shenzhen, Guangdong, China).

### 5.2. Cell Culture

The GSCs were isolated from surgical specimens. These specimens were diagnosed as World Health Organization (WHO) grade IV glioblastoma multiforme. The isolation and cell expansion processes were undertaken following previous reports [30,50]. All the clinical information of the patients was confidential, and the data were analyzed anonymously. Investigations were conducted in accordance with the Declaration of Helsinki ethical standards and the protocol was approved by the Ethics Board of Kunming Institute of Zoology, Chinese Academy of Sciences, China. Isolated GSCs were detected by soft agar colony formation test and analyzed expression profile of GSCs to conduct genetic relationship tree in glioblastoma cells. The character of GSCs was verified through above tests. The U87 cell line was purchased from the Conservation Genetics Chinese Academy of Sciences (CAS) Kunming Cell Bank (Kunming, Yunnan, China), and the T98G cell line was gifted from Iavarone’s lab (Columbia University, New York, NY, USA) [36]. The two cell lines were maintained in DMEM supplemented with 10% FBS and 1% P/S. All the cells were incubated in humidified atmosphere with 5% CO_2_ at 37 °C. 

### 5.3. Cytotoxicity Activity

The cytotoxicity of 3*α*-acetonyltabersonine was quantified via a MTS assay by generating 11-point dose–response curves. Briefly, 2 × 10^4^ cells per well were plated in 96-well plates and treated with the test compound for 72 h. Then, a 10% MTS solution was added to each well. The samples were incubated at 37 °C for 0.5 h at least. The optical density (OD) was measured at 490 nm and detected using a spectrum plate reader, and the IC_50_ values were computed using GraphPad Prism 5 software (version 5.01, GraphPad Software, Inc., San Diego, CA, USA, 2007)) [51].

### 5.4. DNA Proliferation Assay

DNA replication was measured by Click-iT EdU Imaging Kits. The experimental procedure was according to the manufacturer’s protocol and reference [52]. Random fields were observed by fluorescence microscopy and the proportion of EdU-positive cells (%) was counted as the EdU-positive cell number/the total cell number.

### 5.5. Immunofluorescence

Cells were seeded on laminin-coated glass chamber slides and treated with 3*α*-acetonyltabersonine. When the treatment time was over, the cells were fixed in 4% paraformaldehyde (PFA) for 20 min and permeabilized with Triton X-100. Then, 10% goat serum (for cleaved-Caspase-3) or 5% BSA (for γ-H2AX) was used as blocking buffer and subsequently incubated with the primary antibodies. The cells were washed with phosphate buffer saline containing Tween-20 (PBST) and incubated with the corresponding secondary antibodies. The cells were then counterstained with DAPI to visualize the nucleus and mounted on glass slides using Aqua-poly. 

### 5.6. MMP Measurement 

The MMP was detected using JC-1. JC-1 was thawed and reconstituted in culture medium by diluting the reagent 1:10 to generate solution in advance. The cells were treated with or without the test compound, and 10% JC-1 solution was added into the culture medium. After the cells were loaded with JC-1 at 37 °C in the dark for at least 15 min, the cells were immediately visualized using a fluorescence microscope. 

### 5.7. Apoptosis Measurement

Cell apoptosis was analyzed by Annexin-V/PI apoptotic assay. Cells seeded in 6-well plates were incubated at different concentrations of 3*α*-acetonyltabersonine for 16 h. At the end of the incubation, the cells were harvested, washed twice with cold phosphate buffer saline (PBS), centrifuged at room temperature and resuspended in 250 μL binding buffer. Subsequently, the cells were stained with 5 μL Annexin-V and 10 μL PI for 10 min at room temperature in the dark and were analyzed via FCM using FACS VANTAGE SE (Becton Dickinson, Franklin Lakes, NJ, USA). Win MDI software (version 2.8, Scripps Research Institute, La Jolla, CA, USA, 2000) was used for gating, grouping and computing.

### 5.8. RNA Preparation and RNA-seq 

Total RNA extraction and purification were accomplished using the PureLink RNA Mini Kit (Ambion, Austin, TX, USA) and the TURBO DNA-free Kit (Ambion, Austin, TX, USA), respectively. Transcriptome sequencing was performed by Macrogen China (Shenzhen, Guangdong, China). RNA sequencing libraries were constructed using the Illumina mRNA-Seq Prep Kit (Santiago, CA, USA). Low-quality reads were filtered out using the Perl script IlluQC.pl from GSQCToolkitv2.3.3 with the following parameters: pe-cutOffReadLen4HQ 70 and cutOffQualScore 20 N 5. The clean reads were mapped to the human genome (GRCh38) using tophat-2.0.2 and read-mismatches 2, and the expected fragments per kilobase of transcript per million fragments (FPKM) of the genes were calculated using cufflinks2.02 with max-multi-read-fraction 0.75. Finally, the genes were clustered to KEGG pathways. 

### 5.9. Real-Time PCR

The purified RNA was reverse-transcribed using the Revert-Aid cDNA synthesis kit following the manufacturer’s instructions. The primers for real-time quantitative PCR were specific for human genes (Table 1). The PCR amplification process was accomplished using an Applied Biosystems 7900HT Fast Real-Time PCR system (Foster City, CA, USA) in a 20 μL reaction containing 10 μL of SYBR Select Master Mix, 1 μL of diluted cDNA template and 0.5 μL of each primer (10 μM). The real-time quantitative PCR reactions were conducted using the following cycles: 50 °C for 2 min, 95 °C for 2 min followed 40 cycles at 95 °C for 15 s and 60 °C for 1 min, and then incubated at 12 °C. The relative amount of specific mRNA was normalized to GAPDH, and the fold changes were calculated from three replicates in each sample.

### 5.10. Western Blotting

Cells treated with 3*α*-acetonyltabersonine were washed and scraped off with cold PBS, collected by centrifugation at 5500 revolutions per minute (RPM) for 5 min at 4 °C and harvested in lysis buffer for 20 min with gentle mixing 2–3 times. Then, the cell debris was removed by centrifugation at 14,000 RPM for 30 min at 4 °C, and the supernatants were pipetted out carefully. Protein concentration was determined using the Bradford method. All protein extraction processes were performed on ice, and all the tubes were subjected to pre-cooling. Equal amounts of proteins were separated by sodium dodecyl sulfate polyacrylamide gel electrophoresis (SDS-PAGE) and electro blotted onto nitrocellulose (NC) blotting membranes. Then, the membranes were washed with PBST, blocked for 1 h on the shaker, and incubated with the diluted primary antibodies with gentle agitation overnight at 4 °C. The next day, incubation with secondary antibodies was performed at room temperature, and the blots were developed using the chemiluminescent HRP substrate kit. The band signals were detected using an ImageQuant LAS4000 Biomolecular imager (General Electrics, Uppsala, Uppsala, Sweden) and supplementary software. The relative gray scale values of the protein bands were compared using Image J software (version 1.4.3.67, Broken symmetry, Bethesda, MD, USA, 2006). Antibodies used are listed in Table 2.

### 5.11. Comet Assay

Comet assay was performed according to the standard procedure [57]. The DNA was stained with GelRed, and the comet images were visualized and captured by fluorescence microscopy. Image measurements and records were obtained using the comet assay software project (CASP) (version 1.2.2, СASPlab, Wroclaw, województwo śląskie, Poland, 2004), and at least 50 individual images were evaluated randomly from each sample. 

### 5.12. Plasmid Cleavage Assay

The plasmid pGL3 was tested in a designated sodium phosphate buffer solution [35], and the test was conducted at 37 °C for 16 h with different concentrations of 3*α*-acetonyltabersonine.

### 5.13. DNA Damage Recovery Assay

Two groups of cells were only treated with DMSO or 3*α*-acetonyltabersonine. Another two groups were treated with etoposide initially, but the medium was refreshed and commensurable DMSO or 3*α*-acetonyltabersonine was added. When the treatment was over, the cells of the four groups were collected to conduct the comet assay.

### 5.14. Lentiviral Production

The backbone of the pTomo-Ras-sip53 lentiviral vector was a gift from the Verma lab (The Salk Institute for Biological Studies, San Diego, CA, USA) [58]. We inserted a Human Ras fragment between XbalI and SalI using PCR with the primers (F-Ras-XbaI: CGTATCTAGAACCGCCATGGACTACAAGGACGATG; R-Ras-SaII: TCAAGTCGACTCAGGAGAGCACACACTTGCAGCTC). Two package plasmids, pCMV ∆8.1 and pMD2.G, were co-transfected with polyetherimide (PEI) assistant, and the subsequent process was performed as described [59].

### 5.15. Intracranial Injections and 3α-Acetonyltabersonine Treatment

Six-to-eight-week-old-male C57BL/6 mice were purchased from Shanghai SLAC Laboratory Animal Technology Co., Ltd. and were kept in a specific pathogen free (SPF) environment. All mice were injected under anesthesia with a ketamine-xylazine solution in accordance with the guidelines of the International Agency for Research on Cancer’s Animal Care and Use Committee. An aliquot of 1 μL of purified lentivirus particles was injected 1.6 mm lateral and 1.4 mm anterior into the bregma and 1.75 mm below the skull using a stereotaxic frame. The injection rate was 0.1 μL/min, to avoid intracranial hypertension. The mice were then randomly assigned into two groups 10 days later after injection, and the subsequent treatments were as follows: 2 μL of 20 μM 3*α*-acetonyltabersonine or DMSO was administered using intracranial drug delivery every two days. All animals were provided free access to food and water. All animal care and experimental protocols were approved by the Animal Care and Use Committee of Kunming Institute of Zoology, Chinese Academy of Sciences, China. All the investigations were undertaken in accordance with the ethical standards of national and international guidelines.

### 5.16. Histology and Immunohistochemistry

Tissue preparation and immunohistochemistry procedures were performed as previously reported [36]. Antibodies used in the immunohistochemistry studies are listed in Table 2.

### 5.17. Statistical Analysis

Quantitative results were analyzed by GraphPad Prism 5 software and Excel. The levels of significance in this article are as follows: * *p* < 0.05 and ** *p* < 0.01. Significant difference analysis was conducted using *t* test. 

## Figures and Tables

**Table 1:** Primers Used in Real-time PCR.

Gene Name	Forward Primer	Reverse Primer
HSPA6	CTCCAGCATCCGACAAGAAGC	CTCCAGCATCCGACAAGAAGC [53]
HSPA1B	CCCCATCATCAGCGGACTG	AACACCCTTACAGTATCAAC [54]
HSPA1A	CCCCATCATCAGCGGACTG	GGCAAGTTCAGTACTTCACC [54]
FOS	CAACTTCAT TCCCACGGTCA	TGGCAATCTCGGTCTGCAAA ^a^
JUN	GCGTTAGCATGAGTTGGCAC	CGCATGAGGAACCGCATCGC ^b^
NR4A1	CCCTGAAGTTGTTCCCCTCAC	GCCCTCAAGGTGTGGAGAAG ^c^
GADD45B	ATTGCAACATGACGCTGGAAGAGC	GATGAGCGTGAAGTGGATT [55]
DUSP16	CACACCACCATTACATCATCG	AACAGTCTGAAGAGAGAGAGGC [55]
DUSP10	GCGGCAGTACTTTGAAGAGGCTTT	AGTCATGGTCATCCGAGTGTGCTT [56]
DUSP1	CGAAGCGTTTTCGGCTTCC	CACCCTGATCGTAGAGTGG [56]

^a^ Real Time PCR Primer sets (VHPS-3372); ^b^ RT Primer DB (ID 1034); ^c^ Primer Bank (ID 320202955c2).

**Table 2:** Information of Antibodies.

Name	Product Number	Postscript
cleaved-Caspase-3	CST, 9661	Primary antibody
γ-H2AX	Abcam, ab2893	Primary antibody
p-ERK	CST, 4370	Primary antibody
p-JNK	CST, 4671	Primary antibody
p-ATM	Abcam, 36810	Primary antibody
β-actin	Sigma, A1978	Primary antibody
Flag	Sigma, F2555	Primary antibody
Ki67	Vector, vp-K452	Primary antibody
Goat anti-Rabbit IgG Fc Dylight 488	Abcam, ab98462	Secondary antibody
Goat anti -Rabbit IgG (Cy3)	Abcam, ab6939	Secondary antibody
Goat anti-Mouse IgG (Cy3)	Abcam, ab97035	Secondary antibody
Goat anti-Rabbit IgG peroxidase conjugate	Sigma, A6154	Secondary antibody
Goat anti-Mouse IgG peroxidase conjugate	Sigma, A4416	Secondary antibody
